# The mutational landscape and actionable targets of gallbladder cancer: an ancestry-informed and comparative analysis of a Chilean population

**DOI:** 10.3389/fonc.2025.1658528

**Published:** 2025-10-03

**Authors:** José Ignacio Erices, Evelin González, Marcela Salgado, Carol Barahona-Ponce, Matías Freire, Gonzalo Sepúlveda-Hermosilla, Diego Ampuero, Alejandro Blanco, Valentina Gárate-Calderón, Pablo Báez-Benavides, Camilo Tapia-Valladares, Jessica Toro, Iván Gallegos, Olga Barajas, Mónica Ahumada, Verónica Sanhueza, Loreto Spencer, Gonzalo De Toro, Erik Morales, Lorena Gutiérrez, Fernanda Morales, Arnaldo Marin, Nelson M. Varela, Justo Lorenzo Bermejo, Ricardo Armisén, Katherine Marcelain

**Affiliations:** ^1^ Departamento de Oncología Básico Clínico, Facultad de Medicina, Universidad de Chile, Santiago, Chile; ^2^ Centro de Genética y Genómica, Instituto de Ciencias e Innovación en Medicina, Facultad de Medicina Clínica Alemana, Universidad del Desarrollo, Santiago, Chile; ^3^ Departamento de Tecnología Médica, Facultad de Medicina, Universidad de Chile, Santiago, Chile; ^4^ Statistical Genetics Research Group, Institute of Medical Biometry, Heidelberg University, Heidelberg, Germany; ^5^ Corporación de Fomento de la Producción (CORFO) Center of Excellence in Precision Medicine, Pfizer Chile, Santiago, Chile; ^6^ Centro de Informática Médica y Telemedicina, Facultad de Medicina, Universidad de Chile, Santiago, Chile; ^7^ Centro para la Prevención y el Control del Cáncer, Centro para la Prevención y el Control del Cáncer (CECAN), Universidad de Chile, Santiago, Chile; ^8^ Departamento de Anatomía Patológica, Hospital Clínico de la Universidad de Chile, Santiago, Chile; ^9^ Departamento de Medicina Interna, Hospital Clínico de la Universidad de Chile, Santiago, Chile; ^10^ Department of Pathology, Hospital Padre Hurtado, Santiago, Chile; ^11^ Department of Pathology, Hospital Clínico Regional Guillermo Grant Benavente, Concepción, Chile; ^12^ School of Medical Technology, Universidad Austral de Chile at Puerto Montt, Puerto Montt, Chile; ^13^ Department of Pathology, Hospital Regional de Talca, Talca, Chile; ^14^ Department of Preclinical Sciences, Faculty of Medicine, Universidad Católica del Maule, Talca, Chile; ^15^ Department of Pathology, Hospital San Juan de Dios, Santiago, Chile

**Keywords:** gallbladder cancer, next-generation sequencing (NGS), driver mutations, genetic ancestry, personalized therapies

## Abstract

**Introduction:**

Gallbladder cancer (GBC) is a highly aggressive malignancy with one of the highest incidence rates reported in Chile. Despite its clinical impact, molecular characterization of GBC in Latin American populations remains limited, and the absence of effective targeted therapies underscores the urgent need for new therapeutic strategies.

**Methods:**

We collected 118 tumor samples, of which 56 passed sequencing quality control using the Oncomine™ Comprehensive Assay v1. Somatic variants were identified with ANNOVAR and Cancer Genome Interpreter, and ancestry was inferred using ADMIXTURE and PCA with ancestry-informative markers. Comparative analyses were performed with Japanese, Singaporean, and U.S. cohorts.

**Results:**

A total of 535 somatic mutations were detected in 43 genes, with *TP53* (30%), *TSC2* (29%), and *NOTCH1* (27%) being the most frequently mutated. We identified 121 clinically actionable variants in *ATM, BRCA1/2, EGFR, ERBB2*, and other genes. Exploratory analysis suggested an association between higher Mapuche ancestry and *TP53* mutations. Comparative analyses revealed distinct mutational patterns in the Chilean cohort relative to Asian and U.S. datasets.

**Conclusion:**

This ancestry-informed genomic analysis provides the first comprehensive landscape of Chilean GBC, identifying actionable alterations with potential therapeutic relevance and supporting the development of population-specific precision oncology strategies.

## 1 Introduction

Gallbladder cancer (GBC) is the most frequent, aggressive and lethal malignancy of the biliary tract neoplasms. It presents a poor prognosis and low five-year survival rates, due to late diagnosis and limited treatment option. The regions with the highest incidence and mortality rates include Bolivia, India, China, Japan, Bangladesh, and Chile (1, 2). These differences in incidence have been related to both lifestyle and genetic factors, but some risk factors for GBC have also been identified, such as cholelithiasis, chronic inflammation, advanced age and female sex (2, 3). However, the lack of specific symptoms of GBC makes early diagnosis difficult, which decreases the efficacy of available treatments and the potential for curative interventions (4). In fact, it is estimated that only 20% of GBC cases are detected at stages responsive to curative surgical resection (cholecystectomy) (5). While chemotherapy, utilizing gemcitabine and cisplatin, is an option for patients with unresectable GBC, not all patients respond favorably to this treatment (4, 5). Consequently, developing novel strategies to improve the treatment of GBC and facilitate early diagnosis of the pathology is vital to improving the life expectancy of affected patients.

The advent of next-generation sequencing (NGS) has revolutionized precision medicine, enabled detailed genomic characterization of cancers and identified actionable mutations that guide the development of targeted therapies. In GBC, genomic studies have revealed recurrent somatic mutations in genes like *TP53*, *KRAS*, *SMAD4*, and *ERBB2*, many of which represent promising therapeutic targets due to their roles in oncogenic signaling pathways (6). Large-scale genomic analyses, particularly from regions with high GBC incidence like China, confirm a high prevalence of alterations in genes such as *TP53*, *KRAS*, and *ATM*, often with substantial geographic variation, underscoring the influence of population-specific factors on GBC’s mutational landscape (7). These findings emphasize the imperative of integrating genomic profiling into clinical practice and investigating regional molecular patterns to advance precision medicine in GBC.

The Chilean population is admixed, consisting mainly of European and Amerindian ancestry. The main Amerindian groups in Chile are the Aymaras, located in the north of the country, and the Mapuches, located in the south (8). Despite the high incidence and mortality of GBC in Chile, particularly among women with high Amerindian ancestry, genomic studies in this population remain scarce (2, 8). Those that exist are often limited by small sample sizes and do not adequately consider the unique genetic admixture of the Chilean population, which may influence tumor biology and treatment response. These limitations underscore the pressing need for comprehensive genomic investigations that incorporate ancestry-informed analyses, thereby improving our understanding of GBC pathogenesis and supporting the development of more effective, tailored therapeutic strategies for underserved populations.

To bridge this knowledge gap and identify potential therapeutic targets, our study characterized the genomic landscape of a cohort of Chilean GBC patients. We identified 535 somatic variants across 43 of 56 GBC samples. Notably, 22% of these alterations are reported as predictive markers in clinical guidelines for various cancers. Furthermore, our analysis suggests a possible association between higher Mapuche ancestry and the frequency of *TP53* mutations. These findings are crucial for informing the design of new treatment options and prevention strategies, grounded in the specific genetic background of the Chilean population.

## 2 Materials and methods

### 2.1 Patients and samples

Samples were collected in several sites along the country: Clínica Indisa, Biobanco de Tejidos y Fluidos de la Universidad de Chile, Hospital Padre Hurtado, Hospital Regional de Concepción, Hospital Regional de Talca, Hospital de Puerto Montt, Hospital San Juan de Dios, Instituto Nacional del Cáncer, Hospital Regional de Coquimbo, and Hospital Regional de Arica. Hematoxylin and eosin-stained tissue sections, derived from Formalin-Fixed Paraffin-Embedded (FFPE), underwent a thorough review by two pathologists. The tumor region was delineated. Only specimens with a percentage greater than 10% of tumor content were eligible for inclusion in this study. Areas characterized by significant necrosis and intra-tumoral fibrosis were excluded from the analysis. Unstained tissue sections (5 µm-thick) were prepared, deparaffinized, and the tumor tissue specimen was carefully collected for subsequent DNA extraction.

### 2.2 Library preparation and sequencing

DNA was extracted from paraffin-embedded samples using the RecoverAll™ Total Nucleic Acid Isolation Kit (Thermo Fisher Scientific). A 20 ng DNA input was used for library preparation with the Oncomine™ Comprehensive Assay V1 (OCAv1, Thermo Fisher Scientific), following the manufacturer’s protocol. Library preparation was performed using the Ion Chef™ System, with a final library concentration of 100 pM. Purified DNA was quantified using the Qubit™ dsDNA HS Assay and the Quant-iT™ PicoGreen^®^ dsDNA Reagent Kit (Invitrogen, Thermo Fisher Scientific, Carlsbad, CA, USA). DNA purity was evaluated by measuring the absorbance ratio at 260/280 nm. The DNA integrity and fragmentation status were assessed using the High Sensitivity Genomic DNA Analysis Kit (DNF-488) on a Fragment Analyzer system (Agilent Technologies, formerly Advanced Analytical). Sequencing was conducted on an Ion PGM™ System in single-read mode using the Ion 550 kit-CHEF (Thermo Fisher Scientific). The list of genes analyzed and the sequencing quality metrics for all analyzed samples, including total reads, coverage, and uniformity, are summarized in **Supplementary Tables S1** and **2**. Of the 118 FFPE tumor samples collected, 66 (55.9%) yielded sufficient quantity and quality of DNA to proceed to sequencing. The remaining 52 samples (44.1%) failed pre-sequencing QC due to low DNA yield, high degradation. Many of the samples that failed due to low DNA yield or high degradation were obtained from various pathology centers. In these cases, inconsistencies in pre-analytical processes, such as prolonged fixation times and the use of non-buffered formalin, may have significantly contributed to DNA degradation. Ten samples were excluded during post-sequencing quality control for failing to meet predefined variant calling thresholds (≥3 million total reads and ≥90% on-target rate). The final genomic analysis was therefore performed on the remaining 56 patient samples (**Supplementary Figure S1**).

### 2.3 Bioinformatic sequencing data analysis

The data preprocessing and processing were carried out using the OCAv1 v5.18 DNA workflow, using default parameters of the GRCh37/hg19 reference genome. For alignment and variant calling, stringent parameters were defined. Single Nucleotide Variants (SNV) and Indels required a minimum allele frequency of 0.05 and 0.07. The minimum coverage for a variant to be considered was set at 10x for SNV and Indels. Additionally, the minimum coverage for the variant location was set at 200x. Variant annotation was performed using ANNOVAR (9), including RefGene, GnomAD v2.1.1, ESP6500, 1000 Genomes phase 3, CADD v1.3, dbSNP v150, COSMIC v94, CLINVAR 2021, ICGC28, ABraOM, and Snp138NonFlaged. To enhance the filtering of germline variants in tumor samples, large and local population germline variant databases were interrogated:CSVS (10), GnomAD (overall and population specific); BIPMed (Brazilian Initiative on Precision Medicine); and a Chilean database (3, 11) (variants imputed from genotyping two arrays with 1,313 and 2,249 samples from Chilean individuals, and whole exome sequencing (WES) data from 87 individuals (NCBI dbSNP database under accession code 1062069) (12).

### 2.4 Public databases

Mutation data of GBC samples were extracted from the Memorial Sloan Kattering Clinical Sequencing cohorts (6) through cBioportal (https://www.cbioportal.org, accessed on march 1st 2024). In addition, GBC mutation data from Japanese and Singaporean samples were extracted from the International Cancer Genome Consortium (ICGC) website (downloaded on march 1st 2024). Only somatic protein-affecting variants found in primary tumors were analyzed in all datasets.

### 2.5 Analysis of actionable and driver variants

The mutational landscape of GBC samples was characterized using the R/maftools package (13). To identify actionable and driver variants within the cancer samples, the Cancer Genome Interpreter platform (https://www.cancergenomeinterpreter.org/home) was employed. Variants were labeled according to the level of evidence (A, B, C, and D) obtained from VICC integrated knowledge base, following the AMP/ASCO/CAP guidelines. OncodriveMUT and BoostDM algorithms were used for prediction of driver variants (14). Only predicted drivers that were also annotated as “oncogenic or likely-oncogenic” in OncoKB (oncokb.org) were considered.

### 2.6 Ancestry analysis in patients with gallbladder cancer

Genetic ancestry was evaluated using genomic DNA extracted from peripheral blood samples of 37 individuals. The ancestry analysis was performed using the ADMIXTURE software (15) and Genetic PCA with the Eigenstrat function (16). This approach allows for supervised estimation of individual ancestry components, including African, European, Native American Mapuche and Aymara, using a large number of Single Nucleotide Polymorphism (SNP) markers previously defined as Ancestry informative marker (AIM) (15). The panel for the preselection of ancestry-informative markers and the estimation of individual ancestry proportions in genetically admixed Chileans, which included 63 Aymara individuals (17) and 28 Mapuche individuals (17, 18). Additionally, it comprised 206 Europeans (99 Utah residents with Northern and Western European ancestry [CEU] and 107 Iberians from Spain [IBS]) and 108 African Yoruba from Ibadan, Nigeria (YRI) from the 1000 Genomes Project (17).

### 2.7 Statistical analysis

A Chi-square test was performed to compare mutation frequencies between the Chilean GBC cohort and those from Japan (GBC-JPN), Singapore (GBC-SGN), and the MSK2022 cohort. p-values were adjusted using the Benjamini–Hochberg method to control the false discovery rate (FDR), given the large number of genes tested simultaneously. Pairwise Chi-square tests with Yates’ continuity correction were performed as *post hoc* comparisons in cases where the adjusted p-value (FDR) was below 0.05. Multivariate logistic regression models were adjusted to evaluate the association between ancestry and the presence of variants in target genes. Sex, age at the time of collection, history of cholelithiasis, and GBC histology were included as covariates using the following model:


GEN∼ancestry+covariates.

In this model, each target gene status (mutated/non-mutated) was modeled as a function of each ancestry, grouped as high/low, along with the effect of other covariates mentioned. A significance level of 0.05 was defined as statistically significant. Patients were categorized into two groups using the median of each estimated genetic ancestry (European, Mapuche, Aymara, and African). All statistical analyses were conducted using R 4.2.1 software.

## 3 Results

### 3.1 Clinical characteristics of gallbladder cancer patients

We collected a total of 118 FFPE biopsies from GBC. To account for the high heterogeneity of the Chilean population, samples were gathered from several medical centers throughout the country. Of these, 94 (76.6%) were from female patients and 24 (23.4%) from male patients, respectively, with an average age of 62 years at the time of collection. Seventy-eight patients (66.1%) had a history of cholelithiasis in their clinical history (**Table 1**).

**Table 1:** Clinical characteristics of patients included in the study.

Clinical characteristic	Category	N = 118 (100 %)
Sex	Female	94(76.6%)
	Male	24 (23.4%)
Cholelithiasis	No	38 (32.2,3%)
	Unknown	12 (1.7%)
	Yes	78 (66.1%)
Self-declared Ethnicity	Aymara	4 (3.4.%)
	Chilean	94 (79.8%)
	Mapuche	10 (8,5%)
	European	4 (3.4%)
	Atacameño	1 (0.8%)
	Afroamerican	1 (0.8%)
	Huilliche	1 (0.8%)
	Onas	1 (0.8%)
	Unknown	2 (1.7%)
Histology	Adenocarcinoma	100 (84.9%)
	Adenosqueamous	1 (0.8%)
	High-grade Dysplasia	11 (9.3%)
	Instestinal Type	1 (0.8%)
	Papillary	4 (3.4%)
	Neuroendocrine	1 (0.8%)
Grade	G1 (Well differentiated)	36 (35.7%
	G2 (Moderately differentiated)	43 (35.7%)
	G3 (Poorly differentiated)	8 (8.9%)
	G4 (Undifferentiated)	1 (1.8%)
	Not aplicable*	30 (17.9%)
Age (at collection)	Median (range)	62 (31-85)

*Not applicable: It was not possible to determine the degree of the disease.

In terms of ethnicity, most patients self-declared Chilean (Admixed) (79.8%), followed by Mapuche (8.5%), Aymara (3.4%) and European (3.4%). Genetic ancestry distribution at individual level is provided in **Supplementary Figure S3**. The specific genetic ancestry As for the histological diagnosis of the samples, a varied distribution in this type of cancer was revealed, with adenocarcinoma being the most frequent type (84.9%), while squamous cell carcinomas and high-grade dysplasia were less common. Most patients were diagnosed at G1 and G2 stages at the time of surgery (**Table 1**).

### 3.2 Somatic mutation profile of gallbladder cancer

**Figure 1:**
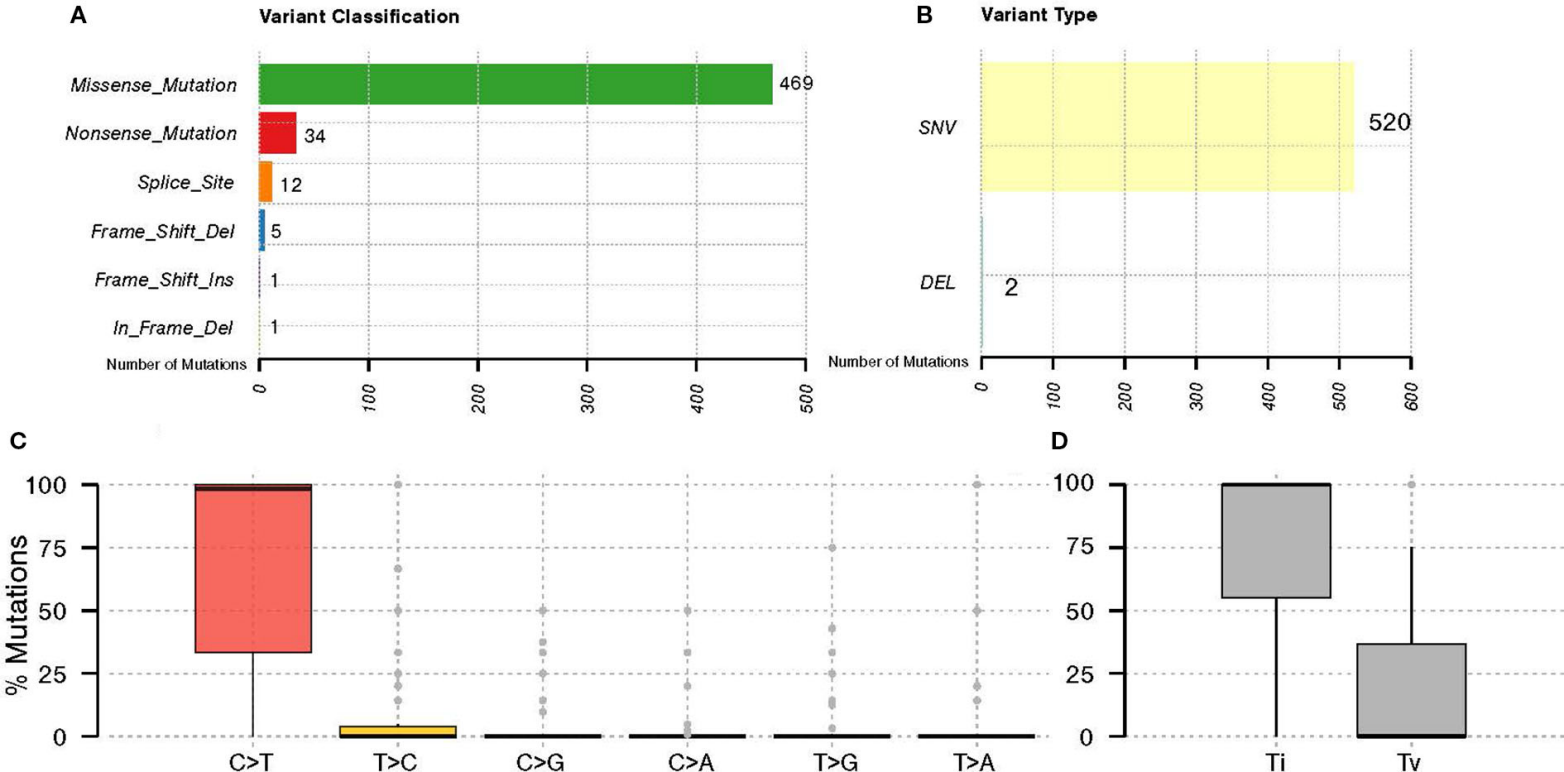
General somatic variant’s classification in GBC. The graph displays the distribution of **(A)** variant classifications, **(B)** types of variants identified in GBC patients, **(C)** nucleotide substitutions of the identified variants, and **(D)** their classification as transitions (Ti) and transversions (Tv).

Just 56 of the 118 samples met all quality control criteria for DNA, library, and sequencing data (**Supplementary Figure S1**). This finding underscores the significant influence of tissue processing and other pre-analytical factors on the successful execution and quality of molecular analyses. We identified 535 somatic mutations across 43 tumor samples. The majority of these somatic variants, 469 (87.66%), were missense mutations, followed by 34 (6.36%) nonsense mutations, 12 (2.24%) splice site mutations, and 5 (0.93%) frameshift deletion mutations. (**Figure 1A**), with the majority falling under the Single Nucleotide Variants (SNV) category (**Figure 1B**). The C > T nucleotide variation was the most frequent substitution among the detected somatic variants. Additionally, transitions (Ti) are more prevalent than transversions (Tv) among the SNVs identified in all genes with mutations (**Figures 1C, D**).

**Figure 2:**
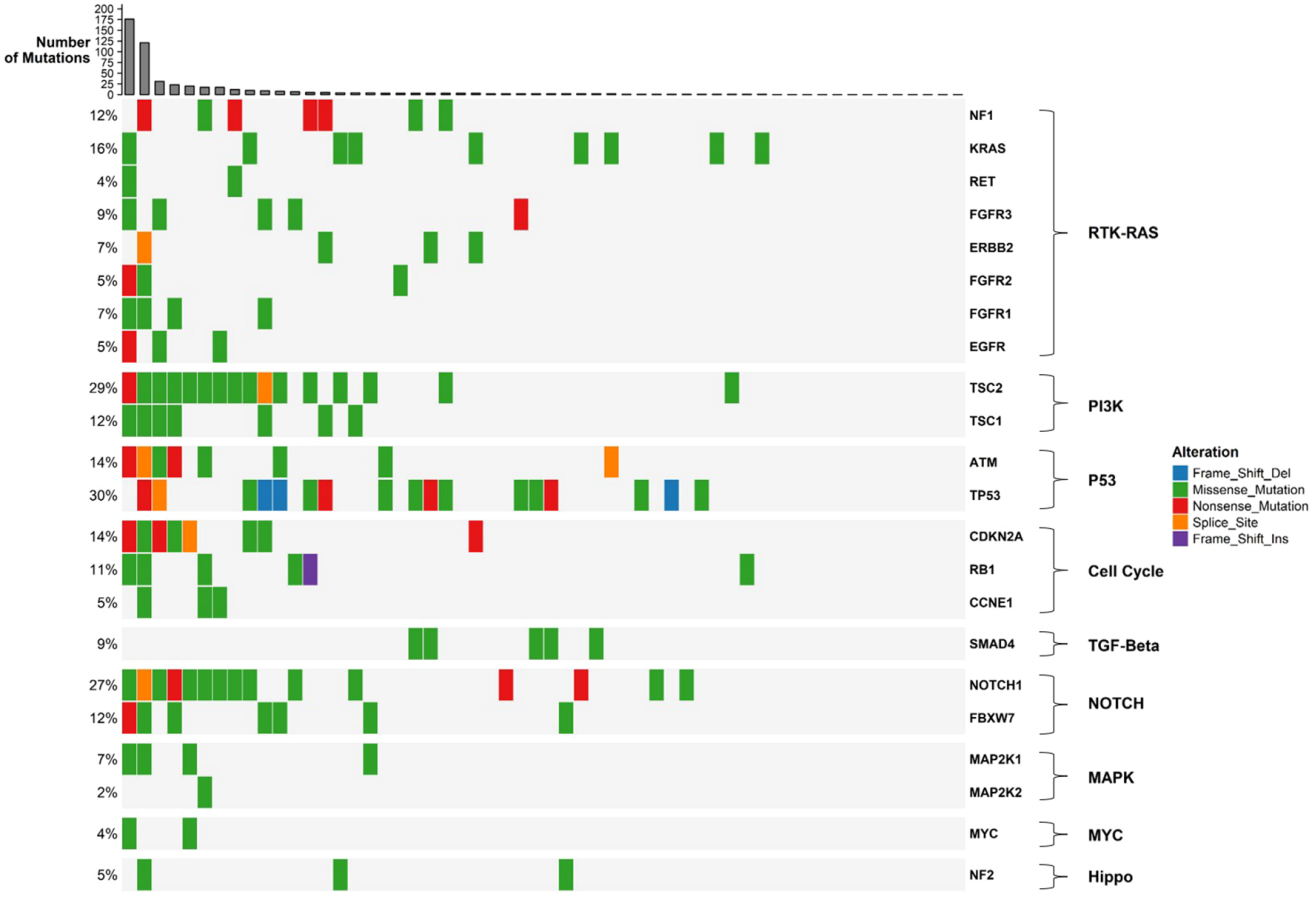
Oncoplot of gallbladder cancer samples grouped by oncogenic signaling pathways. The plot shows somatic mutations in 56 gallbladder cancer samples, with genes grouped according to their associated oncogenic pathways as defined by the TCGA framework. Each colored square represents a mutation type (see legend). Barplots on the top show the number of mutations per sample, while barplots on the right indicate the number and percentage of samples affected for each gene or pathway.

**Figure 3:**
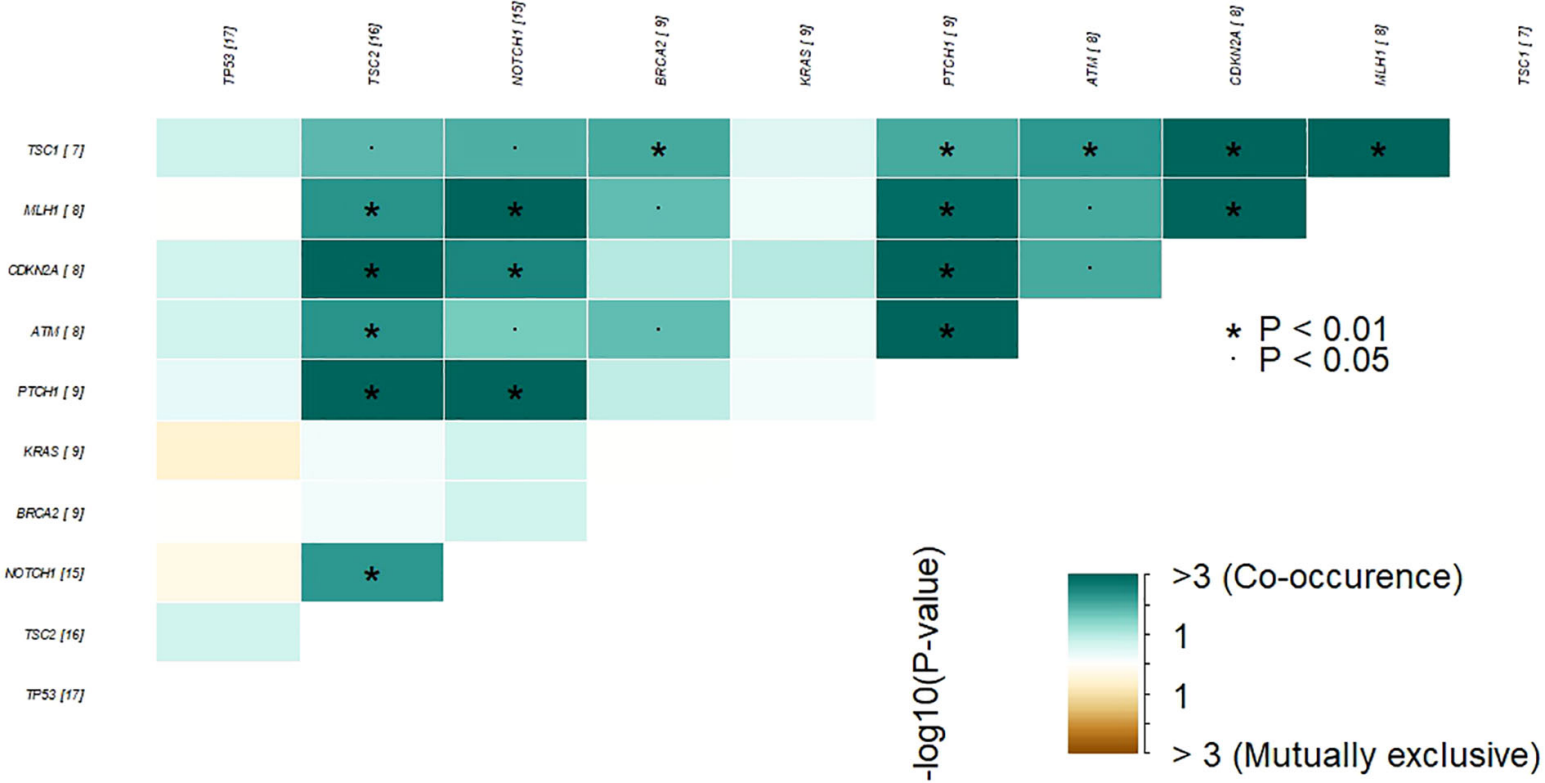
Co-occurrence and mutual exclusivity of the most frequently altered genes in GBC tumor samples. Heatmap showing statistically significant patterns of co-occurrence and mutual exclusivity among the top recurrently mutated genes in our cohort. Color intensity represents the –log10(p-value) for each gene pair. Green tones indicate co-occurrence, while brown tones indicate mutual exclusivity. Asterisks denote statistical significance (P < 0.05; P < 0.01). The numbers in brackets next to each gene indicate the number of patients in our cohort harboring mutations in that gene.

Somatic mutations were identified in 41 out of the 161 genes analyzed. The most frequently mutated genes included TP53 (30%), TSC2 (29%), NOTCH1 (27%), BRCA2 (16%), KRAS (16%), and PTCH1 (16%) (**Figure 2**). The customized oncoplot integrates both gene-level and pathway-level alterations, grouping genes according to their corresponding oncogenic signaling pathways. In this analysis, 2/6 (33.3%) genes in the TP53 pathway and 15/85 (17.7%) genes in the RTK–RAS pathway were altered. Notably, the RTK–RAS pathway was affected in 50% of GBC samples, followed by the TP53 pathway (38%), NOTCH (37%), and PI3K (32%) (**Figure 2**). Among the 10 most mutated genes, several genes show a significant co-occurrence, such as *MLH1*/*TSC2*, *NOTCH1*/*MLH1*, and *NOTCH1*/*TSC2*, among others. Mutations in *TP53* and *KRAS* seem to be mutually exclusive, although this trend did not reach statistical significance (**Figure 3**).

### 3.3 Identification of somatic variants with actionable potential

Given the limited therapeutic options for patients with GBC, identifying targets for therapies that have already demonstrated clinical benefit in other tumor types may enable the inclusion of GBC patients in ongoing or future trials. In our cohort, all 44 patients with somatic variants harbored one or more actionable alteration with supporting Level A evidence. The most frequently altered genes were *KRAS* wildtype (n=35, predictive of response to anti-EGFR therapies such as panitumumab and cetuximab in colorectal cancer), TSC2 (n=16, associated with everolimus in renal angiomyolipoma and giant cell astrocytoma), and BRCA2 (n=9, associated with PARP inhibitors such as rucaparib in ovarian, prostate, and pancreas cancers). Additional Level A alterations were identified in *ATM*, *BRCA1*, *EGFR*, *ERBB2*, *FGFR2*, *FGFR3*, *PTCH1*, *RET*, and *TSC1*, all of which predict responsiveness to specific targeted therapies. Importantly, we detected KRAS mutations (G12D/V/C, G13D, Q61H) associated with resistance to EGFR inhibitors such as cetuximab and panitumumab in colorectal and lung cancer, and responsiveness to KRAS inhibitors sotorasib and adagrasib in colorectal, NSCLC, pancreatic, ampullary, and hepatobiliary cancer (**Table 2** **).**

**Table 2:** Predictive biomarkers found in GBC samples from Chilean patients and supported by level A evidence in various cancers (according to AMP/ASCO/CAP guidelines).

Gene	Alterations	Drugs	Cancer	Effects
*ATM*	W57*, E390K, L508F, E1346K, C1495Y, L1524F, L1562*, A1954V, W2091*, V2671M, L2885F, L2952F	Olaparib (PARP inhibitor)	Prostate adenocarcinoma	Responsive
*BRCA1*	D1872N, E1304K, E1148K	Talazoparib (PARP inhibitor) Niraparib (PARP inhibitor) Olaparib (PARP inhibitor) + Bevacizumab (VEGF mAb inhibitor) Rucaparib (PARP inhibitor)	Breast adenocarcinoma Ovary Prostate adenocarcinoma	Responsive
*BRCA2*	A1109T, K1197X, A2227V, E2239K, R2659K, D2661N, D2733N, E2847K, E3002K, A3297T, P3300L, S3332F, D3341N	Talazoparib (PARP inhibitor), Niraparib (PARP inhibitor) Olaparib (PARP inhibitor) + Bevacizumab (VEGF mAb inhibitor) Rucaparib (PARP inhibitor)	Breast adenocarcinoma Ovary	Responsive
*EGFR*	P772L, R776C, D800N, W898	Afatinib (ERBB2 inhibitor, EGFR inhibitor 2nd gen) Osimertinib (EGFR inhibitor 3d gen) Dacominitinib (EGFR inhibitor) Mobocertinib (EGFR inhibitor) Amivantamab (EGFR mAB inhibitor) Cetuximab (EGFR mAB inhibitor) Erlotinib (EGFR inhibitor 1st gen) Gefitinib (EGFR inhibitor 1st gen)	Non-small lung Colorectal adenocarcinoma Lung	Responsive
*ERBB2*	S310F, R678Q, L755S, E892K, W906*	Trastuzumab deruxtecan-nxki (Antibody-drug onjugate: ERBB2 mAb inhibitor + topoisomerase I inhibitor). Neratinib (ERBB2 inhibitor) + Capecitabine (Chemotherapy) Pertuzumab (ERBB2 inhibitor) + Trastuzumab (ERBB2 inhibitor) + Docetaxel (Chemotherapy) Lapatinib (ERBB2 inhibitor) + Letrozole (Hormone therapy) Fulvestrant (Estrogen receptor antagonist) + Palbociclib (CDK4/6 inhibitor) Margetuximab (ERBB2 inhibitor) Tucatinib (ERBB2 inhibitor) + Trastuzumab (ERBB2 inhibitor) + Capecitabine (chemotherapy	Non-small cell lung Breast adenocarcinoma	Responsive
*FGFR2*	C391Y, D273A, G227E, S215N, W214	Erdafitinib (FGFR inhibitor) Infigratinib (FGFR kinase inhibitor) Pemigatinib (FGFR kinase inhibitor)	Bladder Cholangiocarcinoma	Responsive
*FGFR3*	A18T, Q92*, L379F, L387R, V425M, S426Y, P698S	Erdafitinib (FGFR inhibitor)	Bladder	Responsive
*KRAS*	Q61H, G13D, G12V, G12D, G12C	Cetuximab (EGFR mAb inhibitor) Panitumumab (EGFR mAb inhibitor) EGFR inhibitors	Colorectal adenocarcinoma Lung	Resistant
*PTCH1*	C1038Y, P975S, Q973*, P964S, I941S, A939V, T924M, L834*, Q808*, H520Y, S489L, V477I, G467D, C454Y, S433F, W399*, A174V, R144H, W78*, A70V	Vismodegib (SHH inhibitor)	Basal cell carcinoma Medulloblastoma	Responsive
*RET*	P628S, T754A, S836N, E843K, E902K, D903N, T946I, M984I	Pralsetinib (Tyrosine kinase inhibitor) Selpercatinib (RET kinase inhibitor) Vandetanib (Pan-TK inhibitor)	Thyroid medullary Non-small cell lung	Responsive
*TSC1*	Y1156S, E1128K, R908W, E876K, S832F, R811W, M736I, A728T, G681S, G464S, D405N, D24N	Everolimus (MTOR inhibitor)	Giant cell astrocytoma Renal angiomyolipoma	Responsive
*TSC2*	E79K, W82L, C277Y, W304R, T390M, R458*, T562I, R611Q, P685L, S687F, V868M, Q883*, S981N, G1001R, R1457W, P1521S, D1535N, G1579S, D1598N, R1751C, R1793W, T1804I	Everolimus (MTOR inhibitor)	Giant cell astrocytoma Renal angiomyolipoma	Responsive

The symbol * with the variant nomenclature meaning stop-gain (nonsense) mutation.

### 3.4 Comparative analysis of somatic mutation frequencies and oncogenic variants across diverse gallbladder cancer cohorts

Although genomic data on GBC are available in public repositories, this data is still scarce and has a limited representation of Latin American patients. Given the marked geographical disparities in GBC incidence and mortality, it is plausible to hypothesize that underlying environmental and genomic background may favor the activation of distinct molecular oncogenic mechanisms in high incidence regions. To explore this possibility, first we compared the mutation frequencies in the 41 genes identified in the Chilean cohort with those reported in previously published datasets MSK-IMPACT 2022 cohort, as well as two GBC cohorts from Japan and Singapore (obtained from the International Cancer Genome Consortium (ICGC) (https://dcc.icgc.org/). Despite the limited sample size, we found significant differences in the mutation frequencies of all genes but *ERBB2* across cohorts (**Table 3**).

**Table 3:** Differential somatic mutation frequencies in gallbladder cancer across geographic cohorts.

Gene	GBC-Chile (N=56)	GBC-JPN (N =239)	GBC-SGN (N=71)	MSK 2022 (N= 237)	P-value	FDR
*ARAF*	2/56	2/239	12/71	1/237	6.16 x 10e-13	9.75 x 10e-13
*ATM*	8/56	17/239	32/71	16/237	< 2.2 x 10e-16	4.64 x 10e-16
*BAP1*	7/56	20/239	11/71	3/237	2.86 x 10e-5	3.29 x 10e-5
*BRCA1*	2/56	8/239	22/71	3/237	< 2.2 x 10e-16	4.64 x 10e-16
*BRCA2*	9/56	14/239	26/71	13/237	2.51 x 10e-14	4.54 x 10e-14
*CBL*	4/56	5/239	41/71	3/237	< 2.2 x 10e-16	4.64 x 10e-16
*CCNE1*	3/56	3/239	11/71	1/237	3.02 x 10e-10	3.82 x 10e-10
*CDKN2A*	8/56	6/239	13/71	26/237	3.5 x 10e-5	3.91 x 10e-5
*DDR2*	3/56	3/239	46/71	4/237	< 2.2 x 10e-16	4.64 x 10e-16
*EGFR*	3/56	4/239	60/71	3/237	< 2.2 x 10e-16	4.64 x 10e-16
***ERBB2***	**4/56**	**17/239**	**11/71**	**19/237**	**1.52 x 10e-1**	**1.52 x 10e-1**
*FBXW7*	7/56	10/239	57/71	9/237	< 2.2 x 10e-16	4.64 x 10e-16
*FGFR2*	3/56	6/239	43/71	3/237	< 2.2 x 10e-16	4.64 x 10e-16
*FGFR3*	5/56	7/239	8/71	1/237	1.9 x 10e-5	2.26 x 10e-5
*FGFR4*	5/56	4/239	11/71	4/237	1.34 x 10e-7	1.65 x 10e-7
*GATA2*	3/56	6/239	6/71	1/237	1.5 x 10e-3	1.54 x 10e-3
*JAK3*	3/56	7/239	7/71	1/237	4.29 x 10e-4	4.53 x 10e-4
*KDR*	1/56	14/239	17/71	2/237	3.22 x 10e-12	4.9 x 10e-12
*KRAS*	9/56	40/239	31/71	18/237	2.61 x 10e-11	3.54 x 10e-11
*MAP2K1*	4/56	2/239	42/71	4/237	< 2.2 x 10e-16	4.64 x 10e-16
*MAP2K2*	1/56	1/239	13/71	1/237	3.08 x 10e-13	5.08 x 10e-13
*MAPK1*	2/56	2/239	35/71	2/237	< 2.2 x 10e-16	4.64 x 10e-16
*MSH2*	7/56	5/239	51/71	6/237	< 2.2 x 10e-16	4.64 x 10e-16
*MLH1*	8/56	7/239	14/71	3/237	1.45 x 10e-10	1.96 x 10e-10
*NF1*	7/56	24/239	52/71	10/237	< 2.2 x 10e-16	4.64 x 10e-16
*NF2*	3/56	4/239	38/71	2/237	< 2.2 x 10e-16	4.64 x 10e-16
*NOTCH1*	15/56	10/239	16/71	5/237	3.34 x 10e-14	5.78 x 10e-14
*PTCH1*	9/56	5/239	33/71	1/237	< 2.2 x 10e-16	4.64 x 10e-16
*PTPN11*	2/56	1/239	45/71	2/237	< 2.2 x 10e-16	4.64 x 10e-16
*RB1*	6/56	7/239	45/71	10/237	< 2.2 x 10e-16	4.64 x 10e-16
*RET*	2/56	10/239	21/71	4/237	< 2.2 x 10e-16	4.64 x 10e-16
*SF3B1*	2/56	12/239	18/71	4/237	3.79 x 10e-12	5.53 x 10e-12
*SMAD4*	5/56	22/239	40/71	52/237	< 2.2 x 10e-16	4.64 x 10e-16
*STK11*	5/56	12/239	16/71	22/237	1.49 x 10e-4	1.62 x10e-4
*TERT*	6/56	5/239	20/71	15/237	1 x 10e-11	1.41 x 10e-11
*TP53*	18/56	68/239	36/71	155/237	3.31 x 10e-15	6.62 x 10e-15
*TSC1*	7/56	6/239	22/71	2/237	< 2.2 x 10e-16	4.64 x 10e-16
*TSC2*	16/56	12/239	14/71	3/237	2.05 x 10e-14	3.89 x 10e-14

Frequencies of gene mutations were compared between the Chilean GBC cohort (N = 56) and cohorts from Japan (GBC-JPN, N = 239), Singapore (GBC-SGN, N = 71), and the USA (MSK-IMPACT, N = 237). P-values were determined using a Chi-square test and adjusted for a false discovery rate (FDR). Bold text indicates no statistically significant difference in mutation frequency for that gene across the cohorts.

Bold values indicate statistical significance (p < 0.05).

**Figure 4:**
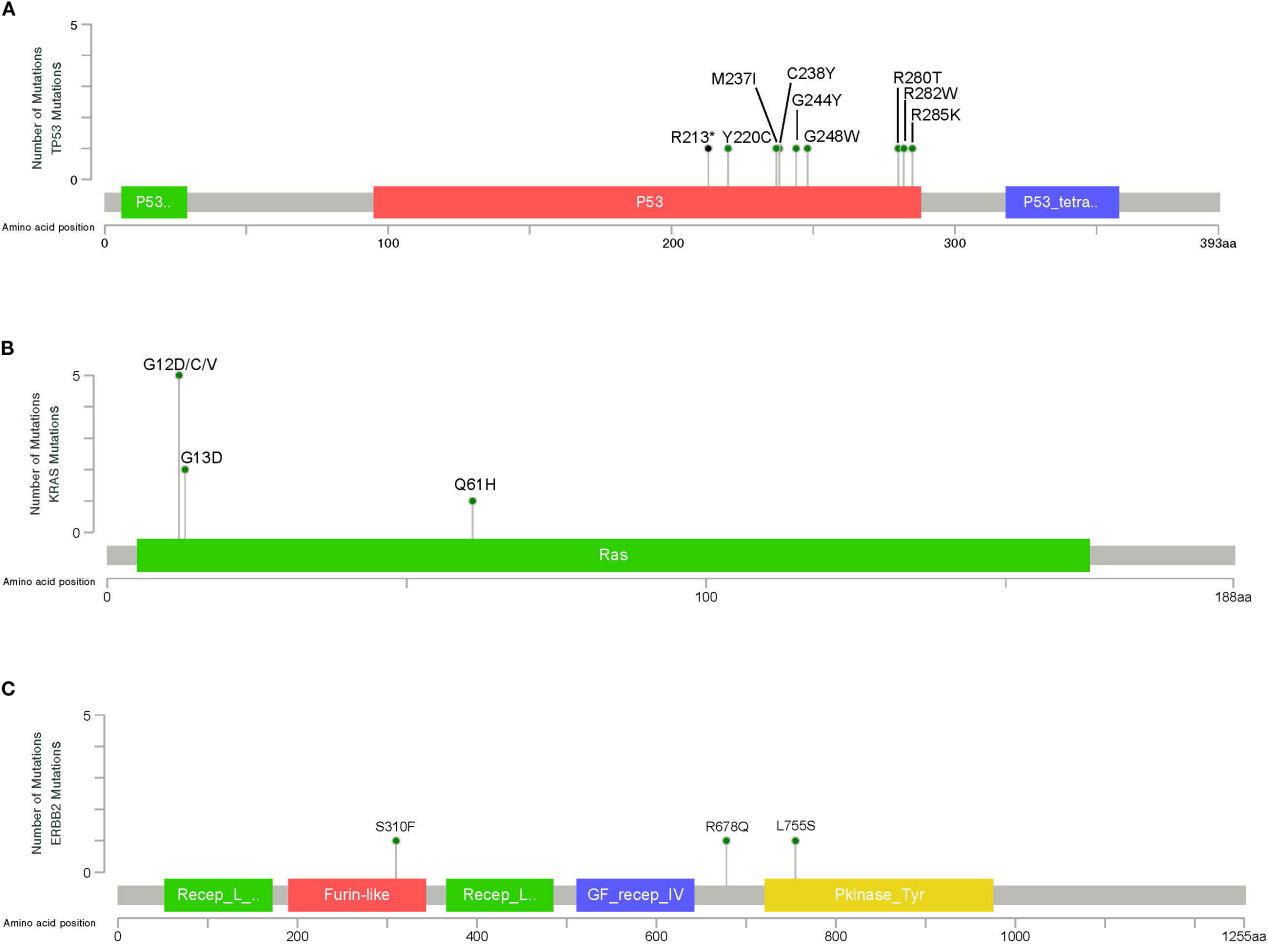
Lollipop plot illustrating the distribution and number of driver mutations in **(A)** *TP53*, **(B)** *KRAS*, and **(C)** *ERBB2*, identified in GBC patients. The gray bar represents the entire protein with the different amino acid positions (aa). The colored boxes are specific functional domains. The vertical axis represents the number of variants per sample. Green and black circles represent missense and nonsense variants, respectively.

Next, we identified variants predicted and annotated as oncogenic and likely-oncogenic in 23 (41.07%) out of the 56 Chilean samples analyzed. These mutations are distributed in 12 genes. *TP53* and *KRAS* exhibit the highest frequencies of oncogenic/likely-oncogenic variants in 18% and 9% of the studied samples, respectively, followed by *ERBB2* (5%) (**Supplementary Figure S2**). *TP53* variants are predominantly missense mutations situated within the p53 DNA-binding domain (**Figure 4A**). *KRAS* recurrent oncogenic G12D/C/V, G13D, and Q61H mutations were found (**Figure 4B**). In *ERBB2* gene, activating S310F, R678Q, and L755S were identified at Furin-like, TMD, and Kinase domains (**Figure 4C**).

To further explore whether the overall differences detected in gene mutation frequencies between GBC from mostly White (MSK.,2022), Asian (GBC-JPN, GBC-SGNAs), and Chilean patients involved key driver mutations, oncogenic variants in the 12 driver genes were identified and compared in all cohorts. Most oncogenic mutations were also found in one or more studies, especially hotspot mutations in *CDKN2A*, *ERBB2*, *SMAD4*, *FBXW7*, *KRAS*, and *TP53*. Nevertheless, differences are also evident, in tumor suppressor genes *ATM* and *TP53*. Notably, in Chilean samples, likely oncogenic EGFR R776C, BRCA2 E3002K, and MLH1 W666* and Q562*, were found. No oncogenic mutations were identified in these genes in the referenced studies (**Table 4**).

**Table 4:** Shared and unique oncogenic driver mutations in Chilean GBC compared to other populations.

Gene	GBC-Chile	GBC-JPN	GBC-SGN	MSK 2022
*ATM*	W57*	R1618*		R35*
*BRCA2*	**E3002K**			
*CDKN2A*	R80*, **P114L**	R58*, R80*, E88*, M531		Q50P, E69*, R80*, D84N, D108N, D108Y, **P114L**
*EGFR*	**R776C**			
*ERBB2*	**S310F**, **R678Q**, **L755S**	G292R, **S310Y**, G660D, **R678Q**, V842I, T862A, L869R.	L869R	T862A, T798I, V777M, **R678Q**, **L755S**, V842I, D759Y, **S310F**, **S310Y**
*FBXW7*	**R479Q**	**R479Q**		R465C, **R479Q**
*KRAS*	**G12V**, **G12D**, **G12C**, **G13D**, **Q61H**	**G12R**, **G12C**, **G12V**, P34L, **G13D**, **G12D**, **G12A**, T58I, **Q61H**, **Q61R**, A146T	G12A, **G12V**, **G12D**, **G13D**, **Q61H**	L19F, **G12C**, **G12A**, **G12R, G12D**, **G13D, Q61H**
*MAP2K1*	E203K	K57N		K57E
*MAPK1*	**E322K**			**E322K**
*MLH1*	W666*, Q562*			
*SMAD4*	**R361C**	E330K, **R361C**, D351N, **R361H**	**R361C**, **R361H**	D537Y, D351H, R445*, **R361S**, **R361H**, **R361C**
*TP53*	R213*, Y220C, **M237I**, C238Y, G244Y, **R248W**, **R280T**, R282W, **E285K**	A138V, R158G, R175H, H179R, H193R, R196*, R213*, Y234C, S241C, S241F, **G244S**, **R248L**, R249S, I254T, **R280T**, **E285K**, E287*, R342*	G135R, R158H, P151, Y163C, R175H, Y236C, **M237I**, **R248L**, **R248W**, V272L, R273H, **E285K**, R306*	Q100*, R110L, K120E, L130F, Q136*, C141Y, R158H, A161T, Y163C, S166*, V172F, R175H, R175C, H179R, P190L, H193Y, L194F, I195T, R196*, Y205C, R213*, Y234C, Y234H, **M237I**, **C238F**, N239S, S241F, G245S, G245D, **R248Q**, **R248W**, R249S, V272L, R273C, R273L, R273H, P278R, **R280K**, **R280T**, **R282W**, **R282Q**, **E285K**, E294*, R306*, R337H, R342*

This table displays oncogenic and likely-oncogenic mutations found in gallbladder cancer (GBC) cohorts from Chile, Japan, Singapore, and the USA (MSK 2022). The gene list was determined by mutations identified in the Chilean cohort. Driver variants were predicted using OncodriveMUT and BoostDM and included only if annotated as "oncogenic" or "likely-oncogenic" in the OncoKB database. (Black Bold: Variant is shared with the Chilean cohort. Green Text: Variant occurs in the same codon as one in another cohort. Red Text: Variant was found only in the Chilean cohort and not in the other referenced studies).

The symbol * with the variant nomenclature meaning stop-gain (nonsense) mutation.

### 3.5 Exploring the relationship between ancestry, somatic mutations, and TP53 in Chilean GB

Genomic studies have provided evidence supporting that Chilean Amerindian genetic ancestry is a risk factor for GBC (3). In fact, a correlation has been established indicating that Chileans with a higher proportion of South Amerindian genetic ancestry (Mapuche, MAP) have a higher risk for GBC (3, 8). With the aim to assess whether this genetic-ancestry-related risk for GBC is also associated with the risk for a specific molecular oncogenic mechanism of disease, we used a panel of Ancestry informative marker (AIM) to estimate the proportion of ancestral genetic backgrounds for 37 out of 43 patients with somatic variants. As expected, European and Mapuche genetic ancestries are predominant in the analyzed individuals (0.493, sd: 0.145; and 0.34, sd: 0,129, respectively), followed by Chilean main North Amerindian Aymara (0.137; sd: 0,221), and African (0.019; sd: 0.16) ancestries (**Supplementary Figure S3**).

A general overview of the somatic mutation´s data suggest that higher proportion of Mapuche ancestry would associate with a lower number of somatic mutations (**Supplementary Figures S4**, **S5**). Although these results are preliminary, this led us to investigate the potential association between Mapuche ancestry and specific gene mutation frequency in *TP53* (the most frequently mutated gene in our restricted cohort). Higher proportion of Mapuche ancestry may be positively associated with mutations in TP53; however, the result is not significant (OR = 6.73, 95% CI: 1.14, 59.6, p = 0.053). In addition, poorly differentiate (G3) tumors showed positive associations regarding the mutational status of *TP53* (p= 0.006) in a multivariate analysis (**Table 5**).

**Table 5:** Multivariate analysis of factors associated with TP53 mutation in GBC.

Characteristic	OR	95% CI	P-value
MAP_proportion			
<0.34525	ref		
>=0.34525	6.73	1.14 (59.6)	0.053
GBC_age			
< 64.77	ref		
>= 64.77	6.39	1.06 (59.8)	0.062
GBC_gender			
Female	ref		
Male	0.92	0.10 (7.29)	>0.900
GBC_cholelithiasis			
No	ref		
Yes	3.59	0.52 (43.5)	0.200
GBC_histology_grade			
G1 (Well differentiated)	ref		
G2 (Moderately Differentiated)	7.55	1.05 (86.7)	0.065
G3 (poorly differentiated)	108	5.60 (6.326)	**0.006**
Not applicable	2.51	0.08 (51.7)	0.500

Results from a multivariate logistic regression model evaluating predictors of TP53 mutation status in gallbladder cancer patients. The model assesses the association with high Mapuche ancestry proportion (≥0.345), age (≥64.77), gender, history of cholelithiasis, and tumor histology grade. Odds Ratios (OR), 95% Confidence Intervals (CI), and p-values are presented. "Ref" indicates the reference group for each variable.

Bold values indicate statistical significance (p < 0.05).

## 4 Discussion

Gallbladder cancer (GBC) is a highly aggressive biliary tract malignancy, consistently associated with poor overall survival (1–3). Despite its relatively low global incidence, GBC exhibits profound geographical disparities. Notably, Chile reports among the highest incidence and mortality rates worldwide, trailing only Bolivia and Bangladesh (1). The absence of specific targeted therapies for GBC underscores the critical need for advanced approaches. Next-generation sequencing (NGS) offers a promising avenue by enabling the identification of actionable and driver genetic variants that could inform treatment strategies.

In recent years, several studies have emphasized the need to characterize the genomic landscape of solid tumors, particularly those that exhibit marked geographic disparities in incidence and mortality, and for which no effective treatment currently exists—as is the case for GBC (1–3). Using molecular records from different geographical regions is essential for understanding population-specific disease characteristics, enabling the development of tailored prevention and management strategies for high-incidence areas like Latin America (19).

In this context, our study investigated the molecular landscape of GBC in a cohort of Chilean patients using a targeted gene panel. Our findings reveal that TP53 mutations are the most common alterations (30%), followed by mutations in TSC2, NOTCH1, BRCA2, KRAS, and PTCH1. This pattern aligns with previous research; for instance, Narayan et al. (20) demonstrated that Chilean GBC patients exhibited the highest frequency of TP53 mutations when compared to cohorts from Japan and the USA. This observation was further supported by the same group’s subsequent targeted sequencing of 233 GBC patients, where TP53 remained the most frequently mutated gene (6). Similarly, Nepal and colleagues (21) reported TP53 as the most mutated gene in a GBC exome analysis that included Chilean individuals. These findings resonate with Yu et al.’s study (7) on 117 Chinese GBC patients, which also identified TP53 as the most frequently mutated gene. Their work suggested that TP53 mutation frequencies in Chinese patients did not differ significantly from Western cohorts, implying a common pathogenic event in GBC development across diverse populations.

### 4.1 Global and local genomic insights in GBC

Beyond our findings, studies in other high-incidence regions, such as by Mishra et al. in India, similarly show a high frequency of TP53 mutations (90.90%), alongside other common alterations like SMAD4, NOTCH1, and ERBB2 (22). This shared high frequency of TP53 mutations between Chilean and Indian GBC patients may stem from common risk factors like gender, bowel habits, and cholelithiasis (23). Chronic inflammation induced by cholelithiasis is a plausible mechanism, potentially triggering TP53 mutations that contribute to GBC development (24). While a common variant in the ABCB4 gene (rs4148808) linked to gallstone disease might play a role in both populations, further studies are needed for verification (25). It is crucial to note that differences in variant allele frequency (VAF) thresholds (e.g., >1% in Mishra et al. vs. >5% in our study) could influence observed mutation frequencies and interpretations of somatic variants.

We also observed a high number of non-synonymous somatic mutations in two patients, both carrying variants in DNA repair genes such as MLH1 and MSH2. Alterations in these genes have been associated with increased mutational load and may suggest potential sensitivity to immune checkpoint inhibitor (ICI) therapies (26). Furthermore, the predominance of C > T substitutions in our mutation pattern indicates a possible APOBEC-mediated mutational signature (27), a finding also reported in a whole exome sequencing study including Chilean GBC patients (21). This opens avenues for signature-based therapies.

Our cohort showed a female-to-male ratio of 4:1, consistent with the well-established female predominance in GBC incidence reported in Chile and other high-incidence regions (1–3). This disparity is thought to be largely driven by the higher prevalence of gallstones and chronic cholecystitis among women, potentially influenced by hormonal and metabolic factors (1–3). While sex-related differences in mutational patterns were not statistically evaluated in our series due to sample size constraints, previous studies have suggested that certain genomic alterations in GBC may vary by sex, including recent evidence describing distinct clinical and molecular profiles between men and women (28). However, these differences are often confounded by underlying risk factor distributions rather than direct sex-linked pathogenic mechanisms. Nonetheless, the integration of sex as a biological variable in future genomic studies of GBC could help clarify its role in tumor development and progression.

### 4.2 Actionable targets and therapeutic implications

Given the limited approved targeted therapies for GBC and the reliance on systemic chemotherapy, we explored the actionable potential of genetic alterations in our Chilean cohort. Using the CGI platform, we identified clinically actionable alterations with drug recommendations in genes such as ATM, BRCA1/2, EGFR, ERBB2, FGFR3, KRAS, PTCH1, RET, and TSC1/2. While many of these drugs are established in other solid tumors, their specific utility in GBC is emerging. For instance, panitumumab combined with gemcitabine and oxaliplatin showed promising results in KRAS wild-type biliary tract cancer (29), and the SGNTUC-019 study demonstrated the efficacy of Tucatinib and Trastuzumab in HER2-positive metastatic biliary tract cancer (30). The recent FDA approval of sotorasib, a KRAS G12C inhibitor, further highlights the potential for targeted therapies (31).

Notably, ATM, BRCA1, and BRCA2 mutations are highly significant due to their involvement in the DNA damage response (DDR) pathway. PARP inhibitors (e.g., olaparib, niraparib) have shown efficacy in BRCA-mutated cancers (32), with recent evidence suggesting benefit in GBC patients with BRCA2 alterations (33). Although ATM alterations are less studied in GBC, their reported presence in biliary tract tumors suggests they could serve as predictive biomarkers (34, 35). These findings collectively underscore the potential for implementing personalized therapies for Chilean GBC patients by repurposing existing drugs based on specific genomic alterations.

The recurrent involvement of RTK-RAS, PI3K, and TP53 pathways in our cohort underscores their central role in the biology of BTC. Alterations in the RTK-RAS pathway, including KRAS mutations, are known to drive tumor proliferation and survival, and may influence sensitivity to targeted agents or MEK inhibitors (36). Similarly, PI3K pathway activation, through mutations in PIK3CA or upstream receptor tyrosine kinases, has been implicated in resistance to standard chemotherapy, and PI3K/AKT/mTOR inhibitors are currently being evaluated in BTC clinical trials (37). *TP53* inactivation, the most frequent event in our cohort, is not only associated with loss of cell cycle control and genomic instability but has also been linked to reduced sensitivity to gemcitabine-based chemotherapy in BTC. This highlights p53 as a potential therapeutic target, with different strategies depending on mutational status: in *TP53*-mutated BTC, direct reactivation of mutant p53 or synthetic lethality approaches targeting cell cycle checkpoint proteins (e.g., Chk1, ATR, Wee1) may be promising; in wild-type p53 BTC, inhibition of its negative regulators (e.g., MDM2, WIP1) could enhance p53 activity (38). Such pathway-level characterization in GBC not only improves our understanding of tumor biology but also informs the rational design of targeted and combination therapies, particularly in the context of overcoming chemoresistance.

### 4.3 Shared genomic drivers and regional differences

Using computational algorithms, we identified driver variants in *TP53*, *KRAS*, and *ERBB2*, genes frequently mutated in other biliary tract tumors (39). TP53 variants, often influenced by external mutagens, include R280 (associated with aristolochic acid exposure) (40, 41) and R248 (linked to tobacco smoke in lung cancer) (42). The recurrence of R248W and R282W variants may stem from increased CpG site methylation during inflammation, a process contributing to GBC tumorigenesis (43, 44).

Our comparison with international cohorts revealed that ERBB2 was the only gene without significant mutation frequency differences across our Chilean cohort and those from the USA, Singapore, and Japan (45, 46). This consistent frequency, also reported by Nepal et al. (21) and Bitter et al. (47), and, importantly, the frequency of ERBB2 alterations was also found to be comparable between Chilean and U.S. cohorts (48). It has been reported that GBC patients harboring ERBB2 alterations exhibit improved overall survival. Thus, ERBB2 may represent a broadly applicable prognostic and therapeutic biomarker across diverse populations (48).

### 4.4 Ancestry, mutational signatures, and therapeutic opportunities

Beyond common drivers, our study uncovered distinct patterns. Two patients exhibited hypermutated tumors, potentially linked to variants in DNA repair genes (MLH1, MSH2) and suggesting responsiveness to immune checkpoint inhibitor therapies (26). The predominance of C > T substitutions in our cohort also points to an APOBEC-mediated mutational signature (27), consistent with previous findings in Chilean GBC patients (21), opening avenues for signature-based therapeutic strategies.

Furthermore, we explored the association between Mapuche ancestry and TP53 mutation likelihood. While not statistically significant, our exploratory analysis suggested a possible association between higher Mapuche ancestry and TP53 mutation status (OR = 6.73, p = 0.053). While this trend did not reach statistical significance, it may reflect underlying biological differences warranting further investigation. This observation should be considered hypothesis-generating, and validation in larger, independent GBC cohorts will be necessary to confirm or refute this potential ancestry-related effect. This aligns with prior research linking Mapuche ancestry to GBC risk variants (e.g., in ABCG8, TRAF3, ABCB1, ABCB4 genes) and increased susceptibility to gallstones, a known inducer of TP53 mutations via chronic inflammation (24, 25, 49, 50). Such observations underscore the influence of genetic ancestry on tumor profiles, as seen in pan-cancer analyses (51).

These results suggest that there may be tumor profiles associated with genetic ancestry. Although the results are preliminary, they indicate that patients with greater Mapuche ancestry may have a lower mutation rate, which could reflect biological differences in the tumorigenesis of this type of malignant tumor, supporting the need to incorporate genomic studies in GBC.

Recently, Zhu et al., reported overall similar mutation profiles in a Chilean vs US GBC cohorts. However, the immune profiles were highly distinct, characterized by higher densities of T cells and PD-1, but lower macrophages in tumors from Chilean patients (52). Although these clinically relevant differences may reflect lifestyle and/or environmental factor dissimilarities between the 2 countries, their findings reinforce the idea that the genetic origin of patients could modulate cancer biology and response to treatment, thus supporting the development of precision medicine strategies that incorporate ancestry, especially in underrepresented populations such as Chileans.

### 4.5 Actionable targets and future directions

Given the scarcity of approved GBC therapies, identification of actionable alterations (e.g., ATM, BRCA1/2, EGFR, ERBB2, FGFR3, KRAS, PTCH1, RET, TSC1/2) in Chilean patients provide a rationale for personalized treatment, particularly as specific therapies for KRAS and HER2-positive BTC are emerging (29–31). Mutations in ATM and BRCA1/2, crucial for DNA damage response, highlight the potential for PARP inhibitors, with promising results already seen in a GBC patient with a BRCA2 mutation (32, 33).

Despite these insights, our study has limitations. For instance, the NGS assay used (Oncomine Comprehensive Assay v1 (OCAv1), although it covers a panel of 161 clinically relevant cancer genes, it does not detect alterations in, for example, ARID1A, which is frequently mutated in GBC, including in Chileans (21, 52, 53).

In addition, although this assay is designed to detect a certain number of structural variants, only a few samples passed QC for RNA sequencing. Same with CNV. This could lead to underestimation of clinically relevant events such as ERBB2 amplification (49). The future application of broader sequencing approaches, such as whole-exome sequencing (WES) or whole-genome sequencing (WGS), in Chilean GBC cohorts would allow for a more comprehensive characterization of the mutational spectrum, the discovery of novel prognostic or therapeutic biomarkers, and the strengthening of analyses by integrating the ancestral component.

Nevertheless, challenges with FFPE sample quality have to be urgently addressed to further advance in a comprehensive and reliable genomic characterization of GBC in the country. Optimizing biopsy handling and preanalytical conditions are crucial to minimize sample attrition and enhance genomic profiling (54–56). Future work should also consider liquid biopsies (cfDNA) as a complementary strategy, given their concordance with tissue mutations and potential for non-invasive detection of actionable variants (57).

In conclusion, we identified actionable and driver genetic variants that may inform tumor response in Chilean GBC patients, laying the groundwork for personalized GBC therapies. Future research should leverage comprehensive genomic methodologies, such as whole-exome sequencing (WES), whole-genome sequencing (WGS), and liquid biopsies, to overcome current limitations and further elucidate GBC’s complex genetic and environmental interactions.

## Data Availability

The data presented in the study are deposited in the Zenodo repository, accession number DOI: https://doi.org/10.5281/zenodo.17051169.
