# Supplementary material for: The mutational landscape and actionable targets of gallbladder cancer: an ancestry-informed and comparative analysis of a Chilean population
Source: Front Oncol. 2025 Oct 3;15:1658528. doi: 10.3389/fonc.2025.1658528 (PMC12531073; doi:10.3389/fonc.2025.1658528)
Supplement: Supplementary file 1 [file Table1.docx]

| Full lenght | | | | | | |
| --- | --- | --- | --- | --- | --- | --- |
| *ATM* | *BAP1* | *BRCA1* | *BRCA2* | *CDKN2A* | *FBXW7* | *MSH2* |
| *MSH6* | *NF1* | *NF2* | *NOTHC1* | *PIKER1* | *PTCH1* | *PTEN* |
| *RBL1* | *SMARCB1* | *STK11* | *TP53* | *TSC1* | *TSC2* |  |
| Hotspot | | | | | | |
| *AKT1* | *ALK* | *AR* | *ARAF* | *BRAF* | *BTK* | *CBL* |
| *CDK4* | *CHEK2* | *CSF1R* | *CTNNB1* | *DDR2* | *EGFR* | *ERBB2* |
| *ERBB3* | *ERBB4* | *ESR1* | *EZH2* | *FGFR1* | *FGFR2* | *FLT3* |
| *FOXL2* | *GATA2* | *GNA11* | *GNA1* | *GNAS* | *HNF1A* | *HRAS* |
| *IDH1* | *IDH2* | *JAK1* | *JAK2* | *KRAS* | *MAGOH* | *MAP2K1* |
| *MAP2K2* | *MAPK1* | *MAX* | *MED12* | *MET* | *MTOR* | *MYD88* |
| *NFE2L2* | *NRAS* | *PDGFRA* | *PP2R1A* | *PTPN11* | *RAC1* | *RAF1* |
| *RET* | *RHEB* | *RHOA* | *SF3B1* | *SMO* | *SPOP* | *SRC* |
| *STAT3* | *U2AF1* | *XPO1* |  |  |  |  |

Supplementary table 1. List of genes explored using the OCAv1 panel for targeted sequencing. The table shows the genes assessed in two categories: (i) Full length, which includes genes fully sequenced to identify mutations, and (ii) Hotspot, which includes genes analyzed in specific regions known to harbor recurrent mutations in cancer.
