# Supplementary material for: The mutational landscape and actionable targets of gallbladder cancer: an ancestry-informed and comparative analysis of a Chilean population
Source: Front Oncol. 2025 Oct 3;15:1658528. doi: 10.3389/fonc.2025.1658528 (PMC12531073; doi:10.3389/fonc.2025.1658528)
Supplement: Supplementary file 2 [file Table2.docx]

Supplementary table 2. Sequencing quality metrics for the analyzed samples. The data preprocessing and processing were carried out using the OCAv1. The DNA workflow, using default parameters and the hg19 (GRCh37) genome reference. The table includes the sample identifier (Sample ID), total number of sequenced reads, total number of covered targeted bases, percentage of reads on target (% On target), median coverage per targeted base, and uniformity percentage (% Uniformity). These parameters were used to assess the quality and consistency of the sequencing data across all samples.

| Sample ID | Total number of sequenced reads | Total number of  uniquely mapped non-  duplicate reads | Total number of covered targeted bases | %On target | Median coverage for amplicon per targeted base | Mean Deapth | % Uniformity | Percentage of  targeted bases with  coverage ≥200 |
| --- | --- | --- | --- | --- | --- | --- | --- | --- |
| 10010145_DNA | 4286133 | 4226077 | 4010547 | 94,90 | 104 (70-160) | 1152 | 93.53 | 94,79 |
| 10010148_DNA | 9520619 | 9412876 | 9124841 | 96.94 | 106 (70-160) | 2656 | 96.51 | 98,49 |
| 10010149_DNA | 4834663 | 4768759 | 4585638 | 96.16 | 107 (75-170) | 1342 | 97.92 | 98,12 |
| 10010151_DNA | 5114649 | 5043774 | 4830422 | 95.77 | 106 (70-165) | 1410 | 98.08 | 98,55 |
| 10010152_DNA | 10130496 | 10007365 | 9686126 | 96.89 | 108 (65-155) | 2871 | 97.38 | 98,84 |
| 10010153_DNA | 4711155 | 4655868 | 4440766 | 95.38 | 104 (80-160) | 1274 | 97.50 | 98,02 |
| 10010156_DNA | 8681479 | 8611934 | 8246787 | 95.76 | 102 (70-160) | 2336 | 96.17 | 98,62 |
| 10010158_DNA | 4201920 | 4171108 | 4023867 | 96.47 | 97 (55-145) | 1087 | 95.80 | 96,14 |
| 10010159_DNA | 3554137 | 3499073 | 3261485 | 93.21 | 99 (70-165) | 905.6 | 94.30 | 92,33 |
| 10010161_DNA | 2928054 | 2839083 | 2640347 | 93.00 | 89 (75-140) | 676.5 | 68.20 | 56,20 |
| 10010203_DNA | 5048563 | 4983355 | 4675881 | 93.83 | 104 (60-160) | 1347 | 92.87 | 96,06 |
| 10010204_DNA | 9193708 | 8988228 | 8626002 | 95.97 | 104 (25-150) | 2472 | 96.10 | 98,41 |
| 10010205_DNA | 4940168 | 4887118 | 4641295 | 94.97 | 104 (60-165) | 1333 | 93.71 | 96,06 |
| 10010206_DNA | 5775293 | 5693179 | 5433570 | 95.44 | 93 (80-150) | 1416 | 64.65 | 69,45 |
| 10010207_DNA | 3217581 | 3172376 | 3015343 | 95.05 | 89 (70-130) | 759.5 | 66.59 | 61,04 |
| 10010254_DNA | 9326070 | 9211035 | 8861936 | 96.21 | 102 (60-160) | 2518 | 95.85 | 98,49 |
| 10010288_DNA | 4121036 | 4087277 | 3986321 | 97.53 | 97 (70-150) | 1078 | 90.57 | 91,27 |
| 10010289_DNA | 3098207 | 3076748 | 2990906 | 97.21 | 98 (25-160) | 812.7 | 83.63 | 79,11 |
| 10010291_DNA | 3989883 | 3963715 | 3793275 | 95.70 | 97 (70-145) | 1025 | 94.94 | 95,13 |
| 10010292_DNA | 4276344 | 4244445 | 4124327 | 97.17 | 92 (65-140) | 1093 | 89.33 | 90,40 |
| 10010296_DNA | 3872648 | 3841415 | 3653953 | 95.12 | 96 (75-150) | 985.6 | 97.05 | 96,88 |
| 10010302_DNA | 9673979 | 9549559 | 9145612 | 95.77 | 104 (25-160) | 2628 | 95.50 | 98,41 |
| 10010304_DNA | 8888677 | 8714946 | 8417766 | 96.59 | 105 (25-160) | 2412 | 87.80 | 95,98 |
| 10010305_DNA | 8382835 | 8277705 | 8023579 | 96.93 | 107 (70-160) | 2346 | 91.71 | 98,76 |
| 10010306_DNA | 9876201 | 9805182 | 9591429 | 97.82 | 110 (55-160) | 2840 | 89.15 | 97,70 |
| 10010309_DNA | 8729265 | 8612556 | 8404993 | 97.59 | 106 (25-155) | 2383 | 85.54 | 94,39 |
| 10010310_DNA | 4131466 | 4109451 | 3997673 | 97.28 | 98 (70-145) | 1087 | 95.39 | 95,58 |
| 10010345_DNA | 6344270 | 6064024 | 5298137 | 87.37 | 87 (25-150) | 1357 | 79.12 | 85,48 |
| 10010024_DNA | 3549922 | 3533833 | 3481532 | 98.52 | 114 (25-160) | 1380 | 82.98 | - |
| 10010028_DNA | 4514033 | 4151729 | 3926705 | 94.58 | 60 (25-145) | 1352 | 81.12 | - |
| 10010029_DNA | 24609466 | 24509725 | 24080804 | 98.25 | 113 (70-160) | 9669 | 81.03 | - |
| 10010030_DNA | 24476716 | 24156392 | 23492091 | 97.25 | 106 (25-225) | 8730 | 59.96 | - |
| 10010032_DNA | 20007020 | 19839967 | 19552287 | 98.55 | 109 (25-230) | 7304 | 69.19 | - |
| 10010034_DNA | 14448407 | 14123237 | 13500402 | 95.59 | 99 (25-220) | 4825 | 53.13 | - |
| 10010035_DNA | 17702397 | 16724701 | 15483728 | 92.58 | 90 (25-165) | 5501 | 58.07 | - |
| 10010036_DNA | 3661445 | 3637902 | 3546590 | 97.49 | 103 (25-150) | 1429 | 94.83 | - |
| 10010039_DNA | 3594358 | 3549306 | 3301209 | 93.01 | 107 (30-230) | 1246 | 75.26 | - |
| 10010040_DNA | 3721560 | 3686799 | 3439783 | 93.30 | 108 (25-230) | 1353 | 82.48 | - |
| 10010042_DNA | 4589439 | 4562865 | 4271297 | 93.61 | 123 (50-250) | 1656 | 82.20 | - |
| 10010081_DNA | 3185116 | 3164200 | 2878156 | 90.96 | 119 (25-130) | 1120 | 83.24 | - |
| 10010082_DNA | 20355042 | 20295216 | 19767540 | 97.40 | 106 (70-155) | 8111 | 93.16 | - |
| 10010086_DNA | 3694252 | 3675434 | 3604865 | 98.08 | 109 (25-160) | 1525 | 90.26 | - |
| 10010087_DNA | 11434020 | 11390764 | 11035372 | 96.88 | 107 (50-155) | 4515 | 93.02 | - |
| 10010092_DNA | 33163235 | 33058592 | 32327997 | 97.79 | 113 (25-240) | 13667 | 94.38 | - |
| 10010093_DNA | 10619383 | 10592635 | 10247315 | 96.74 | 104 (25-155) | 4168 | 92.46 | - |
| 10010094_DNA | 13214082 | 13174061 | 129619580 | 98.39 | 108 (25-155) | 5300 | 92.85 | - |
| 10010103_DNA | 19540864 | 19412620 | 18861301 | 97.16 | 102 (25-150) | 7509 | 97.19 | - |
| 10010104_DNA | 5729209 | 5682842 | 5418589 | 95.35 | 102 (25-155) | 2173 | 95.76 | - |
| 10010105_DNA | 4050886 | 4012668 | 3824473 | 95.31 | 100 (25-150) | 1511 | 94.94 | - |
| 10010106_DNA | 13594612 | 13522662 | 12915494 | 95.51 | 106 (25-160) | 5310 | 96.39 | - |
| 10010109_DNA | 9715900 | 9657440 | 9472982 | 98.09 | 104 (25-155) | 3800 | 87.90 | - |
| 10010110_DNA | 15450671 | 15388012 | 14732482 | 95.74 | 100 (25-155) | 5537 | 46.68 | - |
| 10010111_DNA | 4745732 | 4729936 | 4614525 | 97.56 | 106 (25-155) | 1876 | 55.21 | - |
| 10010121_DNA | 11307706 | 11271705 | 11029363 | 97.85 | 112 (25-155) | 4758 | 94.41 | - |
| 10010124_DNA | 8003945 | 7974320 | 7858692 | 98.55 | 108 (25-230) | 3226 | 93.19 | - |
| 10010131_DNA | 11492404 | 11453456 | 11233549 | 98.08 | 113 (25-230) | 4843 | 94.26 | - |
