# Supplementary material for: The mutational landscape and actionable targets of gallbladder cancer: an ancestry-informed and comparative analysis of a Chilean population
Source: Front Oncol. 2025 Oct 3;15:1658528. doi: 10.3389/fonc.2025.1658528 (PMC12531073; doi:10.3389/fonc.2025.1658528)
Supplement: Supplementary file 3 [file DataSheet1.pdf]

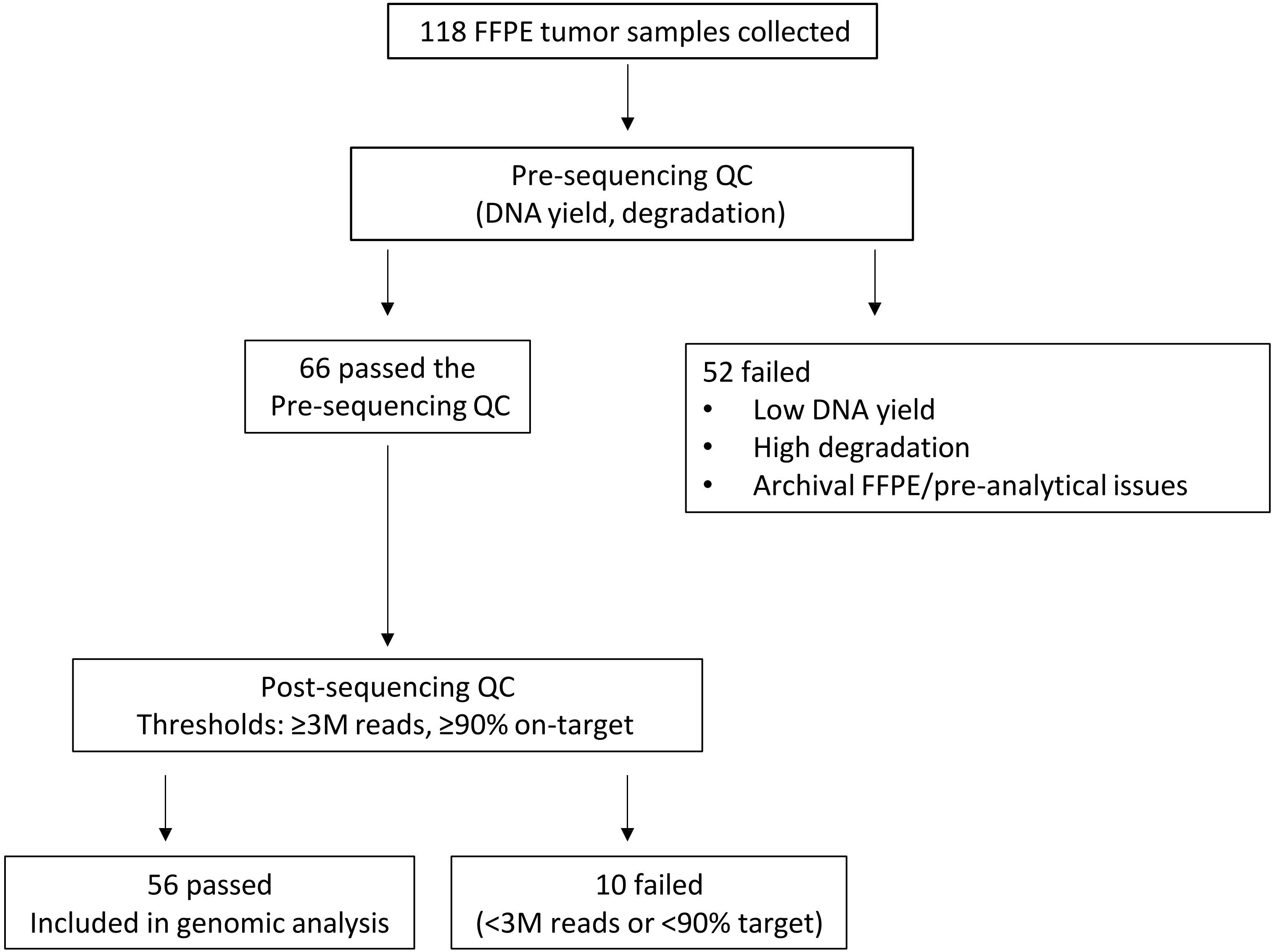

Supplementary Figure 1. Sample flow diagram summarizing the quality control (QC) process applied to the tumor cohort. Of the 118 FFPE tumor samples collected, 52 (44.1%) were excluded during pre-sequencing QC due to low DNA yield, high degradation, or poor library complexity. The remaining 66 samples proceeded to sequencing, from which 10 were further excluded during post-sequencing QC (≥3 million total reads and ≥90% on-target rate). The final genomic analysis was therefore performed on 56 high-quality tumor samples.
