# Supplementary material for: The mutational landscape and actionable targets of gallbladder cancer: an ancestry-informed and comparative analysis of a Chilean population
Source: Front Oncol. 2025 Oct 3;15:1658528. doi: 10.3389/fonc.2025.1658528 (PMC12531073; doi:10.3389/fonc.2025.1658528)
Supplement: Supplementary file 4 [file DataSheet2.pdf]

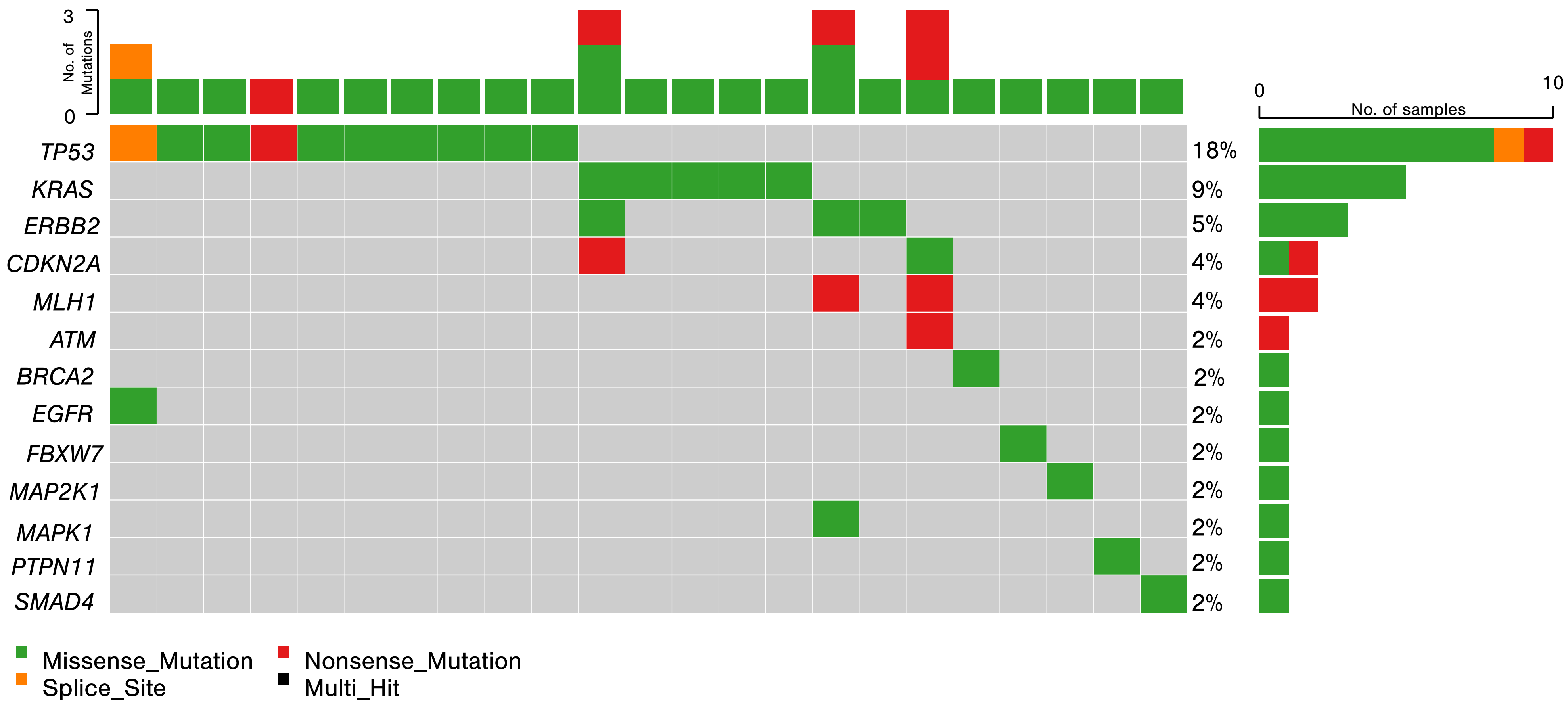

Supplementary Figure 2. Oncoplot showing the distribution of genes with driver variants in the cohort of GBC patients. The oncoplot graph shows the list of genes with variant drivers ordered by mutation frequency in patients diagnosed with GBC. The colored squares indicate mutated genes, while the gray squares indicate non-mutated genes. Variants annotated as Multi_Hit refer to those genes that exhibit multiple variants in the same sample. Only the driver variants were considered, with "oncogenic (predicted and annotated)" classification, by the algorithm Oncodrive MUT and BoostDM available web-site CGI. An analysis restricted to driver mutations shows that a majority are concentrated in the TP53 gene, a finding consistent with the established literature. Mutations were also observed in other known cancer driver genes, such as KRAS and ERBB2, aligning with findings from other cancer studies.
