# Supplementary material for: The mutational landscape and actionable targets of gallbladder cancer: an ancestry-informed and comparative analysis of a Chilean population
Source: Front Oncol. 2025 Oct 3;15:1658528. doi: 10.3389/fonc.2025.1658528 (PMC12531073; doi:10.3389/fonc.2025.1658528)
Supplement: Supplementary file 5 [file DataSheet3.pdf]

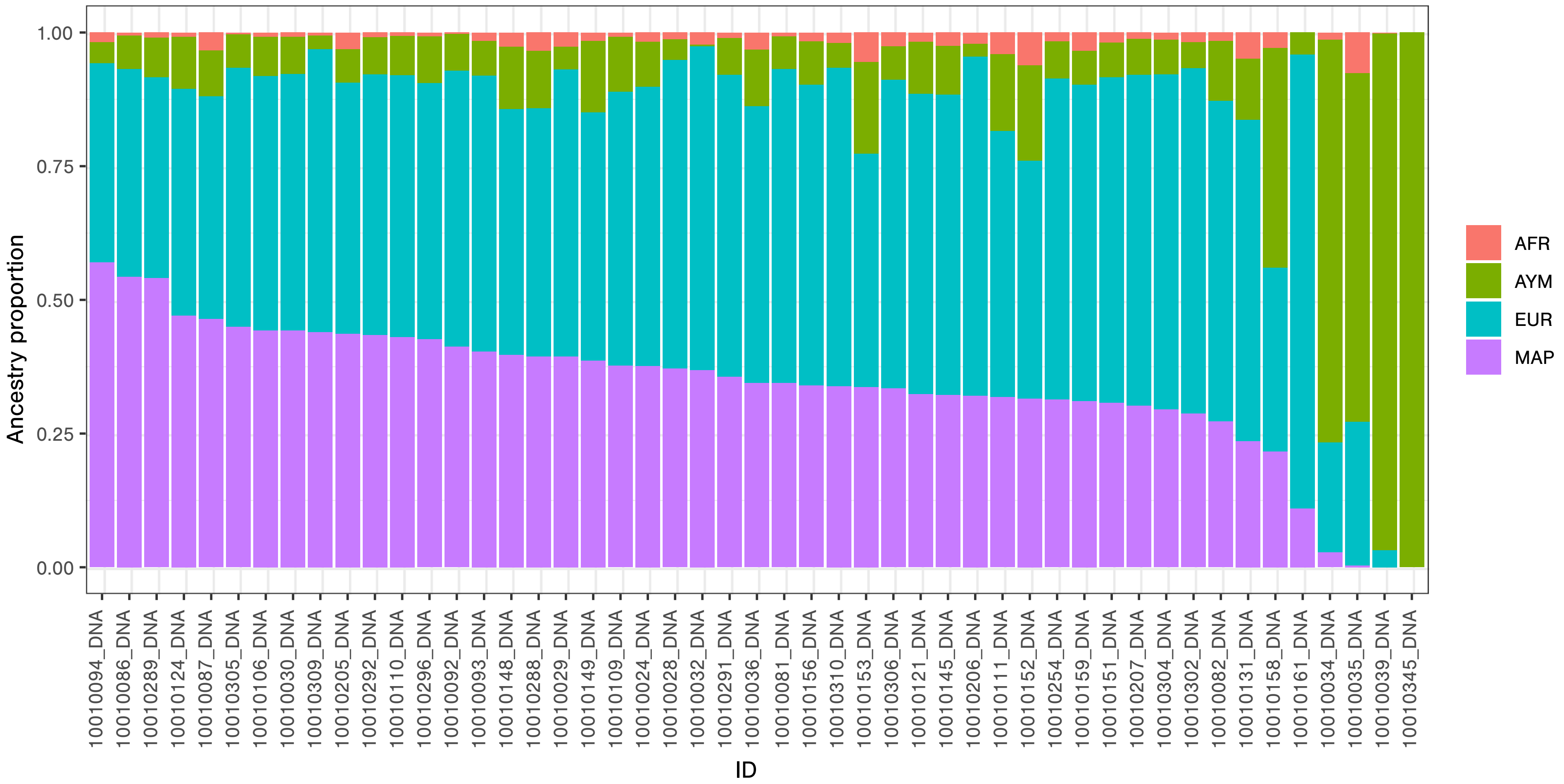

Supplementary Figure 3. Bar graph of individual ancestry proportions. The vertical axis represents the proportion of contribution for each ethnicity, and the horizontal axis represents the individuals analyzed. AFR: African; AYM: Aymara; EUR: European; MAP: Mapuche
Ancestry components were estimated using a panel of Ancestry Informative Markers (AIMs). The reference populations consisted of 63 Aymara and 28 Mapuche individuals for Amerindian ancestry, along with European, African, and Asian data from the 1000 Genomes Project. The plot shows that most individuals have high proportions of Mapuche and European ancestry, consistent with Chile's demographic history. Notably, four individuals exhibit a high percentage of Aymara ancestry.
