# Supplementary material for: The mutational landscape and actionable targets of gallbladder cancer: an ancestry-informed and comparative analysis of a Chilean population
Source: Front Oncol. 2025 Oct 3;15:1658528. doi: 10.3389/fonc.2025.1658528 (PMC12531073; doi:10.3389/fonc.2025.1658528)
Supplement: Supplementary file 6 [file DataSheet4.pdf]

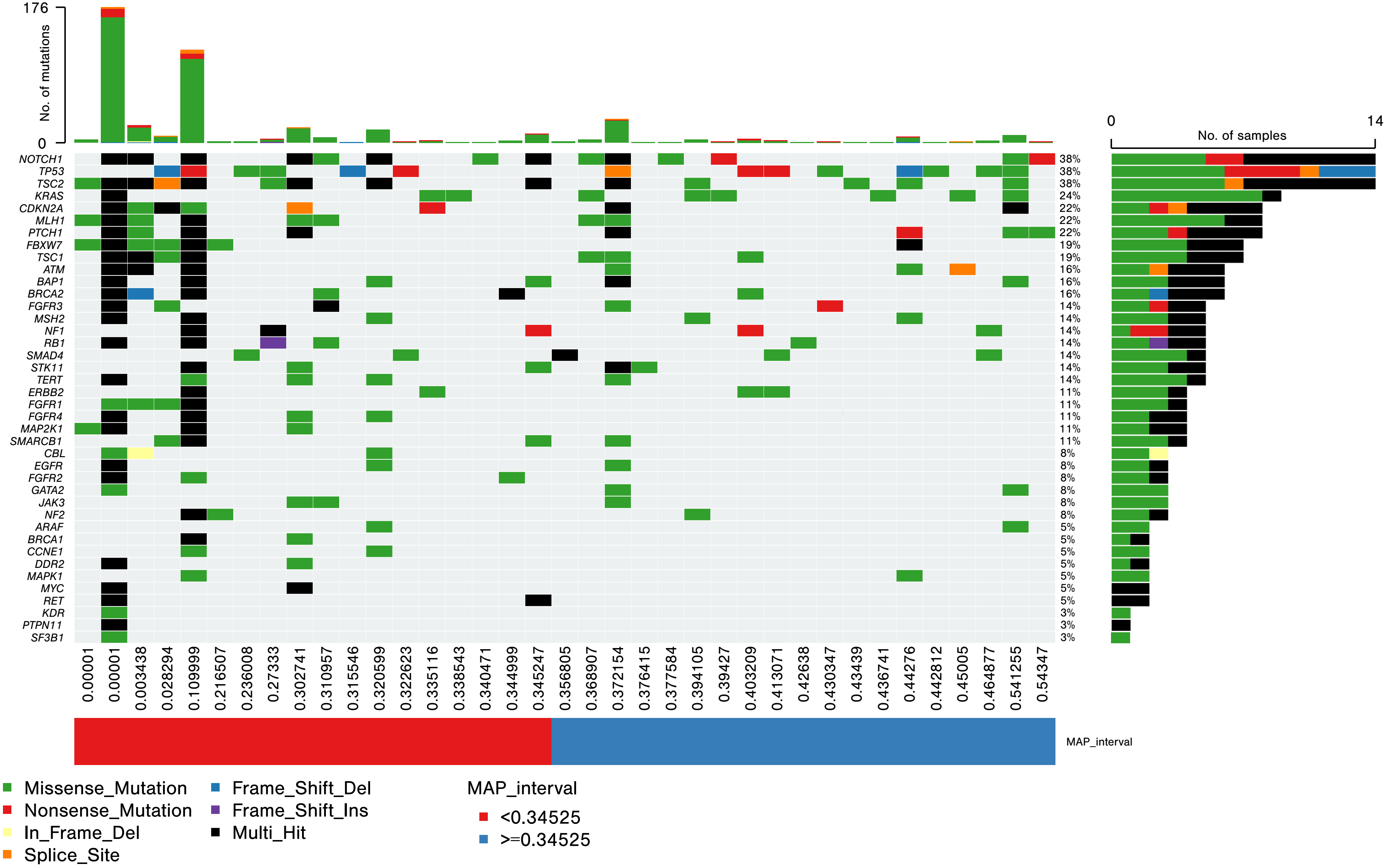

Supplementary Figure 4 . Distribution of somatic variants according to the proportion of mapuche ancestry in patients diagnosed with GBC. The oncoplot illustrates the distribution of somatic variants identified in patients with GBC, ordered from lowest to highest proportion of Mapuche ancestry. Statistical differences were assessed using the non-parametric Mann-Whitney U test. Patients with a proportion of Mapuche ancestry lower than 0.34525 showed a p-value of 0.07.
