# Supplementary material for: The mutational landscape and actionable targets of gallbladder cancer: an ancestry-informed and comparative analysis of a Chilean population
Source: Front Oncol. 2025 Oct 3;15:1658528. doi: 10.3389/fonc.2025.1658528 (PMC12531073; doi:10.3389/fonc.2025.1658528)
Supplement: Supplementary file 7 [file DataSheet5.pdf]

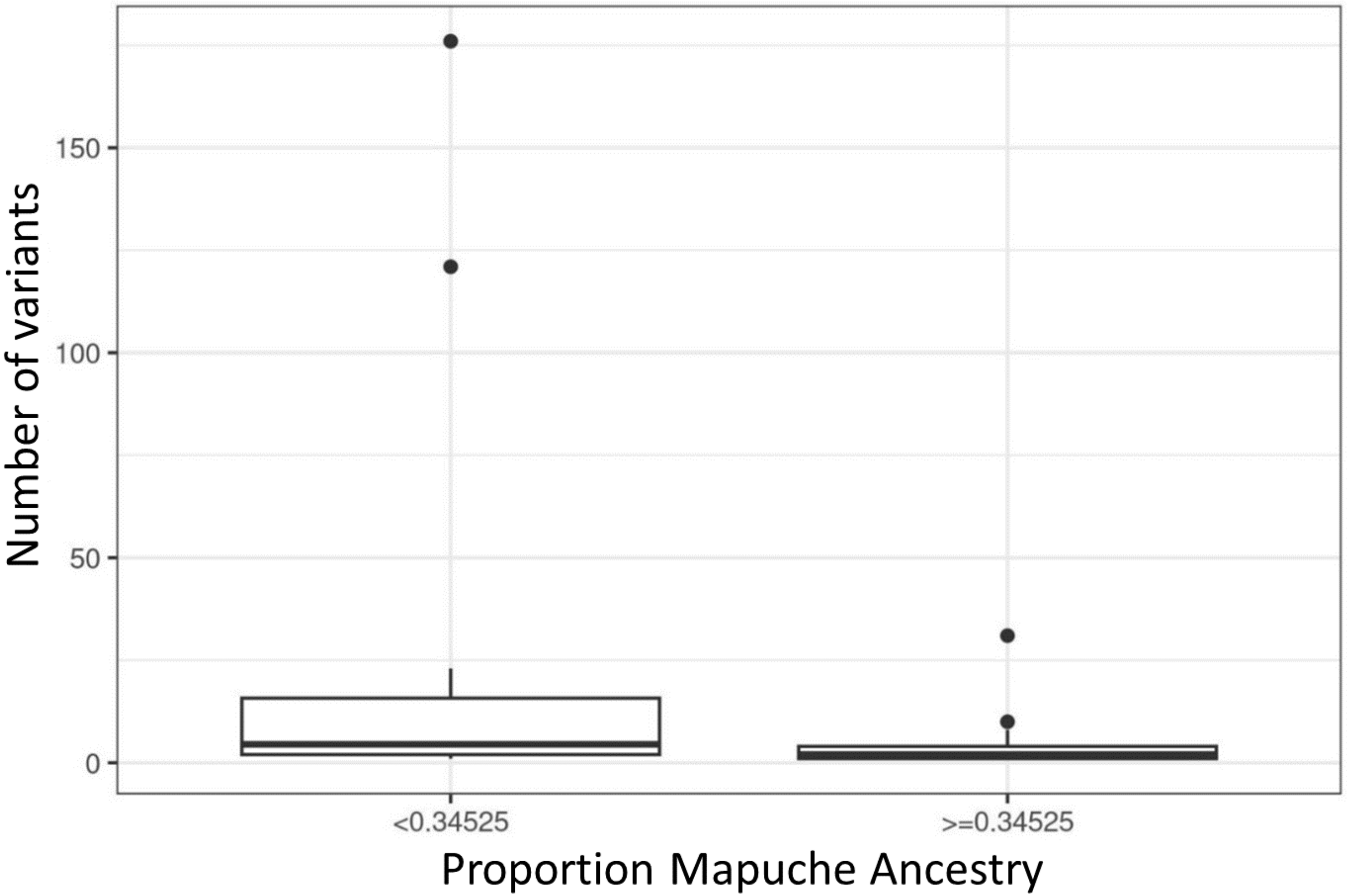

Figure supplementary 5. Distribution of variant counts in patients with high and low proportions of Mapuche ancestry in patients with GBC, according to the group median. The nonparametric Mann-Whitney test was used to determine whether there were significant differences between the measurements of two groups. There was a greater tendency for patients with less Mapuche ancestry to have a higher number of variants, but with a p-value of 0.07
